# Anticoagulant Oligonucleotide–Peptide Conjugates: Identification of Thrombin Aptamer Conjugates with Improved Characteristics

**DOI:** 10.3390/ijms23073820

**Published:** 2022-03-30

**Authors:** Vladimir B. Tsvetkov, Irina V. Varizhuk, Nikolay N. Kurochkin, Sergei A. Surzhikov, Igor P. Smirnov, Andrey A. Stomakhin, Natalia A. Kolganova, Edward N. Timofeev

**Affiliations:** 1 Federal Research and Clinical Center of Physical-Chemical Medicine, 119435 Moscow, Russia; v.b.tsvetkov@gmail.com (V.B.T.); smirnov_i@hotmail.com (I.P.S.); 2 Institute of Biodesign and Complex System Modeling, Sechenov First Moscow State Medical University, 119146 Moscow, Russia; 3 Engelhardt Institute of Molecular Biology, Russian Academy of Sciences, 119991 Moscow, Russia; irina.varizhuk@gmail.com (I.V.V.); nikola.76@mail.ru (N.N.K.); ssergey77@mail.ru (S.A.S.); stomstom@hotmail.com (A.A.S.); natha2006@yandex.ru (N.A.K.)

**Keywords:** thrombin binding aptamer, G-quadruplex, oligonucleotide–peptide conjugate, circular dichroism, anticoagulant activity, molecular dynamics

## Abstract

Oligonucleotide–peptide conjugates (OPCs) are a promising class of biologically active compounds with proven potential for improving nucleic acid therapeutics. OPCs are commonly recognized as an efficient instrument to enhance the cellular delivery of therapeutic nucleic acids. In addition to this application field, OPCs have an as yet unexplored potential for the post-SELEX optimization of DNA aptamers. In this paper, we report the preparation of designer thrombin aptamer OPCs with peptide side chains anchored to a particular thymidine residue of the aptamer. The current conjugation strategy utilizes unmodified short peptides and support-bound protected oligonucleotides with activated carboxyl functionality at the T3 thymine nucleobase. The respective modification of the oligonucleotide strand was implemented using N3-derivatized thymidine phosphoramidite. Aptamer OPCs retained the G-quadruplex architecture of the parent DNA structure and showed minor to moderate stabilization. In a series of five OPCs, conjugates bearing T3–Ser–Phe–Asn (SFN) or T3–Tyr–Trp–Asn (YWN) side chains exhibited considerably improved anticoagulant characteristics. Molecular dynamics studies of the aptamer OPC complexes with thrombin revealed the roles of the amino acid nature and sequence in the peptide subunit in modulating the anticoagulant activity.

## 1. Introduction

**Figure 1:**
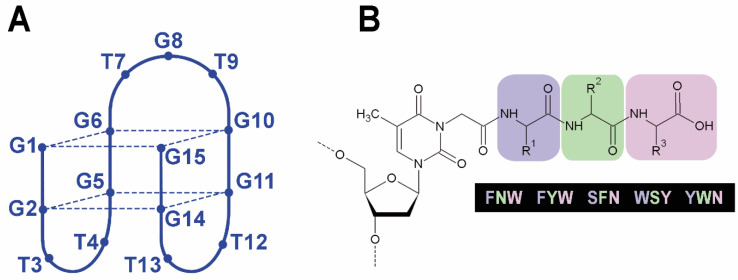
Anti-parallel G-quadruplex formed by TBA (**A**) and peptide subunit of TBA OPCs (**B**). The tripeptide side chain is attached to the thymine base through an N3-carboxymethyl linker that mimics glycine and may be regarded as the fourth invariable amino acid.

Oligonucleotide–peptide conjugates (OPCs) are synthetic constructs that combine peptides and nucleic acids in a single chimeric molecule. Due to the numerous functional roles that peptides and nucleic acids play in living organisms, OPCs have a wide range of potential applications. In particular, OPCs are a promising class of synthetic agents for nucleic acid therapeutics [1,2,3,4]. To a large extent, interest in OPCs is driven by the demand for tools to accomplish the efficient delivery of therapeutic nucleic acids to cells. The versatile functionalities of peptides allow OPCs to address many delivery-related issues, i.e., transport through the cellular membrane, endosomal escape, translocation to the nucleus, or passage through the blood–brain barrier [2,5]. Another aspect of peptide conjugation, which is rarely mentioned with regard to OPCs, is the possibility of enhancing the affinity of the therapeutic nucleic acid component. To a large extent, this application would be most relevant to nucleic acid therapeutics that are not based solely on Watson–Crick interactions. Specifically, DNA (or RNA) aptamers are ideally suited to realize this approach due to the noncanonical mode of interaction of these nucleic acids with their targets. By adding non-nucleoside residues to an aptamer, in particular, peptide side chains, the recognition surface between the aptamer and its target may be potentially expanded. Originally, this strategy was implemented with respect to the thrombin-binding aptamer (TBA) and a variety of chemically diverse groups in a side chain of the aptamer [6]. It was demonstrated that TBA, a 15-nt two-layer antithrombotic G-quadruplex GGTTGGTGTGGTTGG (Figure 1), may be conjugated with a variety of chemical substituents at its T3 residue without affecting its anti-parallel architecture [6]. Some modifications were found to be beneficial for the aptamer characteristics. Specifically, it was shown that the bulky 1-β-D-lactopyranosyl side chain at the T3 residue of TBA improves the affinity and anticoagulant activity of the parent TBA. To further develop this approach, in the current study, we verified whether short peptides conjugated with TBA at the residue T3 could generate additional contacts with the protein and thus increase the anticoagulant effect of the aptamer.

Increasing the contact area at the protein–aptamer interface by adding short peptide residues implies that the amino acid composition of a peptide side chain is complementary to amino acid residues of the respective protein surface in terms of intermolecular interactions. For screening studies, it seems reasonable that the peptide subunit should be composed of the amino acid residues most frequently encountered at protein–protein interfaces. A number of studies have attempted to identify hot-spot amino acid residues in protein–protein contact areas [7,8,9,10,11,12,13,14]. Analysis of the amino acid composition at protein–protein interfaces revealed notable differences with respect to the protein surface exposed to the solvent or to the protein interior. Despite variations in the methods used and the objects studied, the results of these studies point to limited sets of amino acid residues preferably found in protein–protein contact areas. In particular, Arg, Tyr, and Trp should be mentioned as the most representative amino acid residues at protein–protein interfaces. Other hot-spot candidates are Ile, Ala, Ser, Asn, Gly, and Phe.

The suggested chemical tools to synthesize OPCs are either post-synthetic conjugation or stepwise solid-phase assembly of peptide–oligonucleotide sequences on a single solid support [4,15,16]. The former approach seems straightforward and easy to implement, since it is not complicated by compatibility issues between protocols for peptide and oligonucleotide syntheses, that are notably different. The post-synthetic conjugation approach requires the presence of specific functional groups in the peptide and oligonucleotide molecules to join the two subunits of an OPC. Although a large variety of synthetic methods have been proposed for post-synthetic conjugation [15], a new synthetic approach was developed here for TBA–peptide chimaeras due to their specific design. It allows the utilization of unmodified peptides for conjugation through their amino group, thus facilitating the screening of a large variety of commercially available peptide sequences.

Attachment of the peptide side chain at the position N3 of the residue T3 in TBA was carried out through activated carboxylic acid functionality at the thymine nucleobase. The respective side chain was introduced into a synthetic oligonucleotide by using a modified thymidine phosphoramidite derivative.

## 2. Results

### 2.1. Synthesis of TBA–Peptide Conjugates

The phosphoramidite building block for the synthesis of oligonucleotides with activated carboxyl functional groups was designed to carry the p-nitrophenyl (pNP) leaving group. Although most bioconjugation techniques suggest N-hydroxysuccinimide (NHS) esters of carboxylic acids as amine-reactive agents, the preparation and purification of nucleoside derivatives combining both NHS and phosphoramidite functions is cumbersome. Additionally, this activation method is poorly suited for the uninterrupted synthesis of oligonucleotides with internal NHS-functionalized nucleotides [6] due to the low hydrolytic stability of these molecules. We found that the pNP group provides a better alternative to NHS derivatives, since the relatively stable pNP-activated nucleoside phosphoramidite can be seamlessly prepared, purified, and utilized in the synthesis of oligonucleotides with activated carboxyl groups for further conjugation with peptides.

**Scheme 1:**
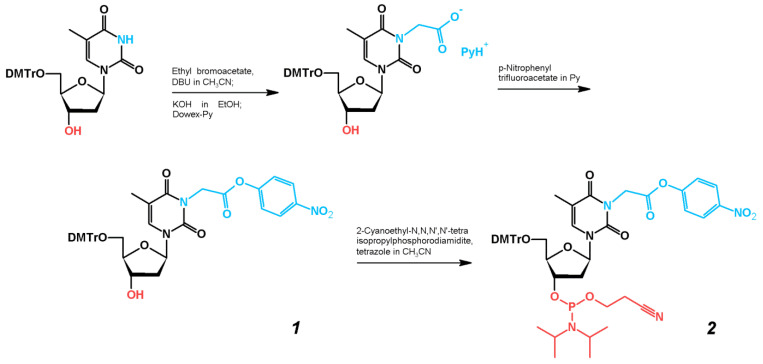
Synthesis of compound **2**.

The preparation of thymidine phosphoramidite derivative **2** with activated carboxylic acid residue at the N3 position of the pyrimidine heterocycle is outlined in Scheme 1. The synthesis of 5′-O-(4,4′-dimethoxytrityl)-N3-carboxymethylthymidine was carried out as described previously by selective N-alkylation of the partially protected thymidine with ethyl bromoacetate in the presence of DBU, followed by alkaline hydrolysis [6,17]. Further activation of the carboxyl group smoothly proceeded upon treatment with an excess of pNP-TFA ester in pyridine [18]. The target phosphoramidite **2** was prepared by a standard reaction of an activated thymidine derivative with 2-cyanoethyl N,N,N′,N′-tetraisopropylphosphorodiamidite in the presence of tetrazole. Importantly, both pNP-activated nucleoside **1** and phosphoramidite **2** could be purified by column chromatography in the presence of 0.3% pyridine without notable damage to the pNP and phosphoramidite functionalities.

The standard cycle of automated oligonucleotide synthesis was modified for compound **2** by increasing the coupling time to 60 s. The preparation of OPC was carried out with controlled-pore glass (CPG)-anchored full-length oligonucleotides prior to ammonia treatment. After examining a few different conjugation protocols using a pNP-modified T_10_ sequence and phenylalanine as a model system, we found that conjugation in sodium carbonate buffer (pH 9.5) for 24 h at 4 °C was the most efficient.

To estimate the hydrolytic stability of the pNP group in the automated oligonucleotide synthesis, we prepared phenylalanine conjugates of two decathymidilates containing a modification either in the 2nd or in the 9th position: 5′-TT*TTTTTTTTTT (T_10_-2) and 5′-TTTTTTTTT*T (T_10_-9). The terminal positions were not selected for modification to avoid preparing a modified CPG substrate and to minimize the expected effect of the bulky 5′-DMTr protecting group on conjugation efficiency. Post-synthetic coupling with phenylalanine in sodium carbonate buffer was followed by a standard ammonia treatment at 55 °C. The analysis of the full-length products by HPLC and MALDI mass spectrometry revealed that the ratio between conjugated and unconjugated oligomers changed, and the efficiency of phenylalanine conjugation dropped from approximately 75% for T_10_-2 to 50% for T_10_-9. This observation suggested the hydrolysis of pNP esters occured, to some extent, in the course of oligonucleotide synthesis, which was most likely associated with the oxidation step by iodine in the Py-THF-H_2_O solution. The hydrolysis of pNP groups is expected to affect the yield of OPCs for long oligonucleotides with modified residues arranged in the proximity of the 3′ end. However, this was not the case for T3-modified TBA analogues.

The design of short peptides for conjugation with TBA was based on a three-residue peptide sequence and the preferable use of aromatic amino acids that could potentially contribute to protein binding through van der Waals interactions. Additionally, we avoided using polar amino acids to prevent their possible strong interaction with the aqueous environment. Taking into account the findings on hot-spot amino acids at protein–protein interfaces [7,8,9,10,11,12,13,14], we selected five tripeptides for conjugation with TBA: SFN, FYW, FNW, YWN, and WSY. The modified residue at the N3 position of thymidine T3 in TBA mimics glycine and may be regarded as the fourth invariable amino acid (Figure 1B).

**Figure 2:**
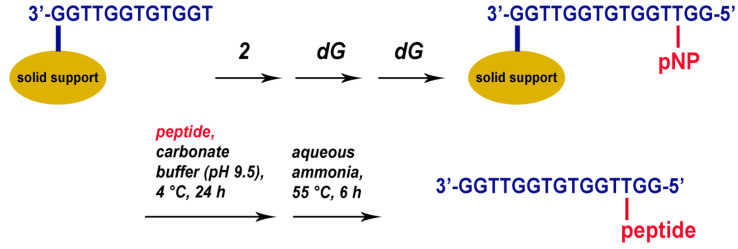
Preparation of TBA OPCs. In the automated oligonucleotide synthesis, phosphoramidite **2** was added at position 3 of TBA. All OPCs were prepared in DMTr-on mode for further purification by reverse-phase HPLC.

Similar to T_10_ models, conjugates of TBA with tripeptides were prepared by reacting a CPG support with an anchored pNP-activated TBA sequence and the respective peptide in sodium carbonate buffer (pH 9.5) at 4 °C for 24 h (Figure 2). To enable reverse-phase purification of TBA OPCs, a T3-modified TBA oligonucleotide was prepared in DMTr-on mode. Notably, cleavage from the support and removal of the protecting groups is partially accomplished in aqueous sodium carbonate at pH 9.5. Nevertheless, the standard ammonia treatment at 55 °C should be applied as a final step before HPLC purification of the crude OPC mixture to ensure complete deprotection. Based on the analysis of HPLC fractions, the highest efficiency of conjugation of 72% was observed for OPC TBA–YWN (Table 1).

Although the current approach was developed specifically for the synthesis of T3-modified TBA, it may be easily adapted for the preparation of a wide variety of OPCs, preferably with a peptide unit arranged in the proximity of the 5′ end of the oligonucleotide. The presence of a single unprotected amino group in the peptide sequence is required to implement the described method for the regioselective preparation of OPCs.

### 2.2. Folding, Stability, and Anticoagulant Activity of TBA–Peptide Conjugates

**Figure 3:**
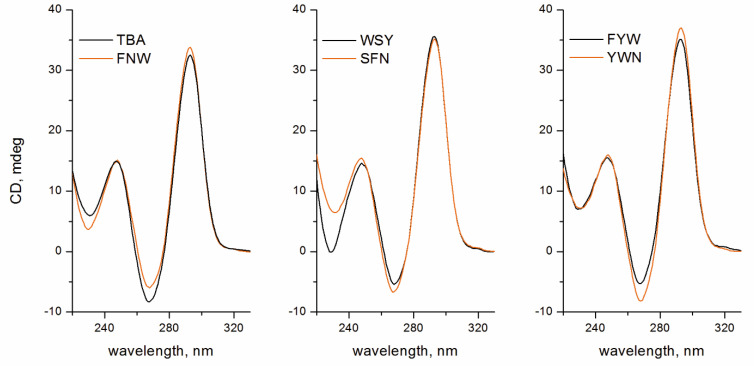
CD spectra of TBA OPCs at 20 °C with an OPC concentration of 5 μM in 10 mM sodium cacodylate (pH 7.2) and 100 mM KCl. All five OPCs are characterized by an antiparallel TBA-like CD pattern.

Since the presence of a peptide unit may potentially affect the folding and stability of the TBA G-quadruplex architecture, we studied TBA OPCs in this respect. Folding of TBA OPCs was inspected by circular dichroism (CD) spectroscopy in 100 mM KCl buffer (Figure 3). The typical pattern of a two-layer antiparallel G-quadruplex featuring a negative band at 268 nm and two positive bands at 248 and 293 nm was reproduced for all studied TBA OPCs. Further study of the thermal stability of folded structures by UV spectroscopy in 100 mM KCl buffer showed that adding a peptide unit to the T3 residue of TBA induced minor to moderate stabilization compared to the parent structure (Table 1 and Figure S1). The *T*_m_ values for TBA OPCs fell within the range of 54.2–57.9 °C. (Table 1). The most notable stabilization effect (+5.4 °C) was detected for TBA–FYW. These findings suggest that the peptide side chain at T3 did not interfere with the TBA architecture. Moreover, depending on the amino acid sequence, the peptide subunit can induce notable stabilization, which should be associated with specific intramolecular interactions.

**Figure 4:**
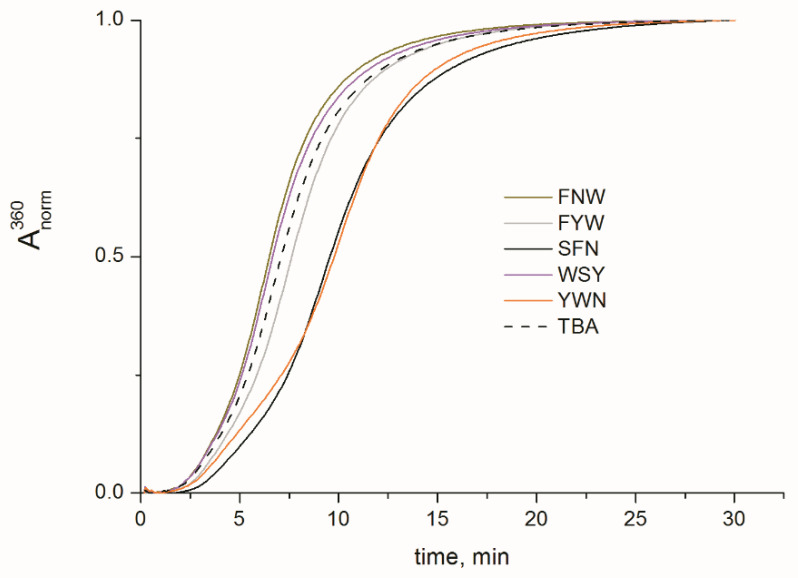
Normalized fibrinogen clotting curves in PBS at 25 °C in the presence of TBA OPCs. TBA–YWN and TBA–SFN demonstrate notably improved anticoagulant activity.

To examine whether any of the added peptide units had an effect on the functional properties of TBA OPCs, their inhibitory characteristics with respect to thrombin were measured. The anticoagulant characteristics of TBA OPCs were evaluated in fibrinogen clotting time tests. Thrombin-induced polymerization of fibrinogen in the presence of TBA OPCs was studied by the increase in absorbance at 360 nm due to light scattering. The clotting curves for five OPCs as well as for unmodified TBA and blank samples are shown in Figure 4. For comparative purposes, we measured the time required to reach 50% of the absorbance maximum (t_1/2_, Table 1). Depending on their amino acid composition, the peptide side chains induced diverse effects on the anticoagulant characteristics of TBA OPCs. TBA variants with the peptide residues FNW and WSY appeared to be less potent thrombin inhibitors than the unmodified aptamer. The modification with the FYW side chain induced a modest increase in clotting time, while considerable enhancement of the anticoagulant characteristics was associated with the tripeptides SFN and YWN. Notable variations in the t_1/2_ values for TBA OPCs clearly point to the additional interactions that emerge in the protein–aptamer complex due to the presence of a peptide unit. To gain insights into the details of the interactions between TBA OPCs and thrombin, we performed molecular dynamics simulations of five thrombin complexes.

### 2.3. Molecular Dynamics Studies of TBA OPC Complexes with Thrombin

**Figure 5:**
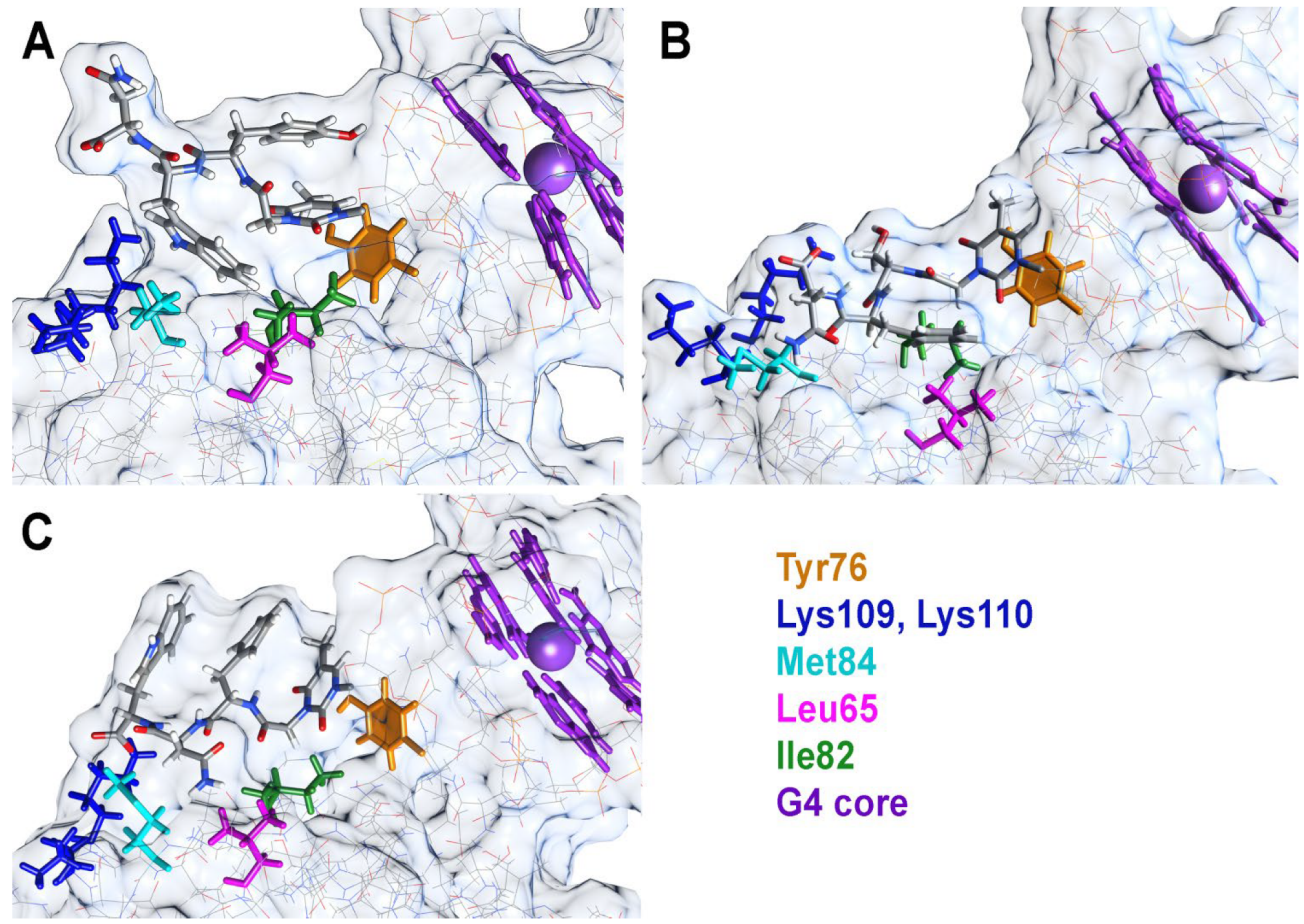
Snapshots of thrombin complexes with TBA–YWN (**A**) and TBA–SFN (**B**) at 80 ns and with TBA–FNW (**C**) at 17 ns. Aromatic residues Tyr76, T3, Phe17, and Trp19 form an extended stacking π-array in the TBA–FNW complex with thrombin at 17 ns.

The previously reported structures PDB 1HAO and 6Z8X were used to generate the starting models of peptide conjugate complexes with thrombin. Peptide residues were optimized before joining the nucleotide and peptide subunits. As reported for closely related modified TBA analogues [6], non-natural T3 residues are invariably located in the region B of thrombin due to the directing role of the unmodified T12 nucleotide. This arrangement brings the modified thymine base of T3 into proximity to Tyr76. Similarly, tripeptide residues in all starting models are located in the region B of the protein. The designation of tri-peptide amino acids refers to residues 17, 18, and 19 from the N- to C-end. The carboxymethyl linker between the T3 pyrimidine base and the peptide unit that mimics glycine was regarded as residue 16 in each conjugate. The total simulation time was 80 ns, which was enough to estimate the dynamics of the peptide unit, T3 base, and thrombin residues in the proximity of the modified fragment (Figure S2). Aside from previously reported TBA–thrombin contacts, additional intermolecular interactions were observed between the peptide modules of conjugates and Lys109, Lys110, Met84, Leu65, and Ile82 of thrombin. Importantly, due to the presence of aromatic amino acids in the peptide module, in most conjugates, we observed intramolecular stacking contacts with the T3 nucleobase. The free carboxylic group at the C-end of the peptide unit tended to stick to the amino groups of either Lys109 or Lys110. This latter interaction did not last long and emerged occasionally during the simulation. Snapshots of TBA OPC complexes with thrombin at selected aspect angles are presented in Figure 5 and Figure S3.

In the most potent thrombin inhibitor, TBA–YWN, Tyr17 stacked with the T3 base most of the time (Figure 5A). This was clearly observed from the dynamics of the angle and distance between the two residues (Figure S4). The Tyr17 aromatic ring was able to flip occasionally, as confirmed by a quick change in the angle between normals from 20–40° to 130–160°. Periodically, the T3 pyrimidine base insignificantly weakened its contacts with Tyr76 (Figure S4), but overall, the stacking system Tyr17–Thy3–Tyr76 was highly stable. The most notable contribution to the additional interactions of TBA–YWN with thrombin should be attributed to Trp18, which is allocated in close proximity to the residues Met84, Leu65, and Ile82. The distance between these amino acids and Trp18 is predominantly within the range of 4–6 Å. The cumulative value of van der Waals and electrostatic interactions was highest for the pair Trp18–Met82, thus pointing to the major role of Met82 in the direct contact with the YWN subunit (Figure S4). The terminal residue Asn19 turned outward from the complex and did not form stable contacts with the protein. Despite this fact, Asn19 may play an important role as a steric barrier between thrombin and fibrinogen. Indeed, this configuration is preserved by highly stable arrangements of Tyr17 and Trp18.

The conjugate TBA–SFN demonstrated virtually the same anticoagulant activity as TBA–YWN. However, its structural organization is notably different from that of TBA–YWN (Figure 5B). Residue Ser17 is not aromatic and does not support stacking with the T3 base. In fact, Ser17 along with Gly16 serves as a linker between TBA and Phe18. It is the latter that formed steady contacts with the protein residues, largely with Ile82 and Leu65 (Figure S5). Interestingly, the dynamics of Phe18–Ile82 interactions affected the stacking between the T3 base and Tyr76. Of all conjugate complexes, only TBA–SFN showed an ability to flip its T3 thymine base, as seen from the profile of angles between normals in the pair T3–Tyr76 (Figure S5). Similar to all other OPCs, the C-terminal residue occasionally interacted with the amino groups Lys109 and Lys110.

In the FYW conjugate, Phe17, Trp19, T3 base, and Tyr76 tended to form a π-stacking system (Figure S6). The T3 base in TBA–FYW formed a highly stable stacking contact with Tyr76. Phe17 added the third stacking layer but showed frequent flipping. After 20 ns of simulation, the average distance between Phe17 and T3 bases stabilized at approximately 5 Å. Trp19 was occasionally involved in the aromatic stack array (Figure S6). In general, this extended π-array was primarily intramolecular in nature and did not greatly increase the affinity of the conjugate to thrombin. Tyr18 is the only residue that contributed to direct binding with thrombin through interactions with Met84, Leu65, and Ile82. However, these interactions did not seem to be optimized due to the Trp19 stacking bias.

TBA–WSY and TBA–FNW are two relatively weak anticoagulants that share important structural features. The aromatic amino acids 17 and 19 tended to form intramolecular π-stacking arrays (Figure 5 and Figure S3), while the non-aromatic amino acids 18, i.e., Ser18 or Asn18, formed weak contacts with thrombin residues. Similar to the TBA–FYW stacking system, Trp17 or Phe17 formed stable pairs with the T3 nucleobase (Figures S7 and S8). Only Phe17 exhibited the ability to flip its aromatic ring. The large aromatic residue Trp17 was unable to rotate and retained the angle between normals within the range of 20–40°. Expectedly, Trp19 in TBA–FNW showed a higher bias for stacking than Tyr19 in TBA–WSY.

The results of molecular dynamics simulations suggest that the amino acid sequence of the tripeptide side chain module at T3 of TBA plays a primary role in additional interactions with the protein and, hence, influences the anticoagulant characteristics of the conjugates. Although TBA–YWN appeared to be the most potent thrombin inhibitor, TBA–SFN OPC generally presents the optimal design of the tripeptide subunit. Since amino acid 18 is involved in major contacts with Met84, Leu65, and Ile82, it should preferably be aromatic or hydrophobic in nature. In contrast, positions 17 and 19 should not be occupied by an aromatic amino acid in order to avoid the formation of an intramolecular stack array. In particular, the C-terminal amino acids should presumably be Asp or Glu to enhance the interactions with the thrombin residues Lys109 and Lys110.

## 3. Materials and Methods

### 3.1. Materials and Instrumentation

Chemical reagents and solvents were purchased from various commercial suppliers and used without further purification. Thin-layer chromatography (TLC) was carried out using Sorbfil aluminum sheets (Imid, Krasnodar, Russia) and visualized by UV absorption or stained with 2% H_2_SO_4_ in EtOH and heated. Column chromatography was performed using silica gel (particle size 0.06–0.2 mm, Sigma–Aldrich, Burlington, MA, USA). Nuclear magnetic resonance spectra were obtained with a Bruker AMX400 spectrometer. MALDI mass spectra were recorded with a 4800 Plus MALDI-TOF mass spectrometer (AB Sciex, Framingham, MA, USA). Research-grade human thrombin from plasma for clotting studies was purchased from Renam (Moscow, Russia). Fibrinogen from human plasma was obtained from Sigma–Aldrich. Standard reagents for automated oligonucleotide synthesis were purchased from Glen Research (Sterling, VA, USA).

### 3.2. Syntheses of Compounds ***1*** and ***2***

5′-O-(4,4′-dimethoxytrityl)-N3-(p-nitrophenoxycarbonylmethyl)thymidine (**1**). p-Nitrophenyl trifluoroacetate (1 g, 4.2 mmol) was added to a stirred solution of 5′-O-(4,4′-dimethoxytrityl)-N3-carboxymethylthymidine triethylammonium salt [6] (1 g, 1.4 mmol) in 20 mL of dry pyridine at 0 °C. The solution was allowed to warm to room temperature (25 °C) for 2 h. The reaction progress was monitored by TLC (DCM/hexane/EtOH 9:10:1, *v*/*v*/*v*). After complete conversion, pyridine was evaporated in vacuo. The residue was diluted with DCM (150 mL), washed with water (100 mL), and dried over anhydrous Na_2_SO_4_. The solvent was evaporated in vacuo. The title compound was purified by column chromatography on silica gel using a gradient of ethanol (0–5%) in a DCM/hexane mixture (1:1, *v*/*v*) containing 0.3% pyridine. Yield 0.67 g (65%). R_f_ 0.5 (DCM/hexane/EtOH 9:10:1, *v*/*v*/*v*). ^1^H NMR (400 MHz, DMSO-d6): δ 8.30–8.35 and 7.42–7.48 (m, 4H, C_6_H_4_NO_2_), 7.68 (d, J 1.1 Hz, 1H, H6), 7.21–7.42 and 6.88–6.95 (m, 13H, DMTr aromatic), 6.28 (t, J 6.7 Hz, 1H, H1′), 5.37 (d, J 4.5 Hz, 1H, 3′-OH), 4.94 (s, 1H, N-CH_2_), 4.34–4.40 (m, 1H, H3′), 3.92–3.97 (m, 1H, H4′), 3.73 (s, 6H, DMTr OCH_3_), 3.19–3.29 (m, 2H, H5′), 2.20–2.37 (m, 2H, H2′), 1.54 (d, J 0.9 Hz, 3H, 5-CH_3_). ^13^C NMR (100 MHz, DMSO-d6): δ 166.5, 162.1, 158.1, 154.7, 150.0, 145.3, 144.6, 135.4, 135.2, 129.7, 127.9, 127.6, 126.8, 125.4, 122.9, 115.7, 113.2, 108.7, 85.9, 85.7, 84.5, 70.3, 63.6, 52.0, 42.2, 12.1; HRMS calcd for C_39_H_37_N_3_O_11_ [(M+Na)^+^]: 746.2323, found: 746.2320.

5′-O-(4,4′-dimethoxytrityl)-N3-(p-nitrophenoxycarbonylmethyl)thymidine-3′-O-[(2-cyanoethyl)-N,N-diisopropyl phosphoramidite] (**2**). Compound **1** (440 mg, 0.6 mmol), pyridine (0.075 mL, 0.9 mmol), and tetrazole (56 mg, 0.8 mmol) were dissolved in 3.5 mL of anhydrous MeCN, and 3–5 beads of 4 Å molecular sieves (4–8 mesh) were added to the solution. The mixture was left under argon for 30 min. Then, 2-cyanoethyl N,N,N′,N′-tetraisopropylphosphorodiamidite (0.23 mL, 0.8 mmol) was added to the solution under intensive stirring. The reaction was allowed to proceed for 30 min at room temperature (25 °C) while monitoring by TLC (DCM/hexane/EtOH 9:20:1, *v*/*v*/*v*). The solution was then diluted with ethyl acetate (100 mL) and washed twice with cold water (100 mL). The organic layer was dried over anhydrous Na_2_SO_4_ and evaporated to a viscous oil. The residue was purified by column chromatography on silica gel using a gradient of ethyl acetate (10–50%) in hexane containing 0.3% pyridine. Yield 0.4 g (71%), mixture of diastereomers. R_f_ 0.7 (DCM/hexane/EtOH 9:10:1, *v*/*v*/*v*); 0.65 and 0.7 (hexane/ethyl acetate 2:1, *v*/*v*). ^1^H NMR (400 MHz, DMSO-d6): δ 8.29–8.35, 7.23–7.47, and 6.87–6.93 (m, 17H, aromatic DMTr and C_6_H_4_NO_2_), 7.69–7.71 (m, 1H, H6), 6.26–6.32 (m, 1H, H1′), 4.88–5.00 (m, 1H, N-CH_2_), 4.57–4.64 (m, 1H, H3′), 4.03–4.12 (m, 1H, H4′), 3.73 (2s, 6H, DMTr OCH_3_), 3.55–3.70 (m, 4H, CH_2_CN and CHNMe_2_), 3.22–3.35 (m, 2H, H5′), 2.65 and 2.77 (2t, J 5.9, CH_2_CN), 2.39–2.47 (m, 2H, H2′), 1.56 and 1.59 (2d, J 0.8 Hz, 3H, 5-CH_3_), 0.99–1.15 (m, 12H, iPr CH_3_). ^13^C NMR (100 MHz, DMSO-d6): δ 167.0, 162.6, 158.7, 155.2, 150.5, 150.1, 145.8, 145.0, 136.6, 135.8, 135.6, 130.2, 128.4, 128.2, 127.3, 125.9, 124.4, 123.4, 119.4, 119.2, 116.4, 113.7, 109.4, 109.3, 86.5, 86.4, 85.5, 85.4, 85.1, 84.7, 73.4, 73.2, 73.1, 72.9, 63.6, 63.5, 58.9, 58.7, 55.5, 43.2, 43.0, 42.7, 24.8, 24.7, 24.6, 20.9, 20.3, 20.2, 12.7. ^31^P NMR (120 MHz, DMSO-d6): δ 147.8 and 147.4. HRMS calcd for C_48_H_54_N_5_O_12_P [(M+Na)^+^]: 946.3399, found: 946.3416.

### 3.3. Automated Oligonucleotide Synthesis and Conjugation

DNA oligomers were synthesized using an ABI 3400 DNA/RNA synthesizer in DMTr-on mode. The coupling time for phosphoramidite **2** was set to 60 s. Support-bound protected oligonucleotides T_10_-2 and T_10_-9 were treated with 1 M D-phenylalanine in a mixture of 0.2 M sodium carbonate (pH 9.5) and DMF (5:1, *v*/*v*, 180 μL) for 24 h at 4 °C. TBA–OPCs were prepared by reacting the support-bound protected modified TBA sequence with the respective peptide (10 mg) in a mixture of 0.2 M sodium carbonate (pH 9.5) and DMF (2:1, *v*/*v*, 150 μL) for 24 h at 4 °C. Partial deprotection of the conjugates was carried out with concentrated ammonia for 6 h at 55 °C. DMTr-protected oligonucleotides were purified by reverse-phase HPLC (Hypersil ODS, 5 μm, 4.6 × 250 mm; 10–50% MeCN in 50 mM TEAA for 30 min). After removal of the 5′ DMTr group, the oligomers were repeatedly purified by reverse-phase HPLC (0–25% MeCN in 50 mM TEAA for 30 min), with the control of the fractions by MALDI mass spectrometry (Figure S9) and denaturing gel electrophoresis. The concentrations of the aptamer OPCs were determined by measuring the UV absorbance at 260 nm. The molar extinction coefficients for aptamer OPCs were calculated using previously reported extinction coefficients for tryptophan, tyrosine, and phenylalanine at 260 nm (3787 M^–1^ cm^–1^, 582 M^–1^ cm^–1^ and 147 M^–1^ cm^–1^) [19].

### 3.4. Ultraviolet Thermal Denaturation

The absorbance-versus-temperature profiles were obtained with a Cary 3500 UV–VIS spectrophotometer equipped with a Peltier cell holder (Agilent Technologies, Santa Clara, CA, USA). The melting experiments were performed at 295 nm in 10 mM sodium cacodylate (pH 7.2) and 100 mM KCl. The heating rate was 0.5 °C/min. The melting points were determined from derivative plots of the melting curves. The oligonucleotide concentration was 5 μM.

### 3.5. Circular Dichroism Spectroscopy

Circular dichroism (CD) measurements were performed at 20 °C with an aptamer concentration of 5 μM in 10 mM sodium cacodylate (pH 7.2) and 100 mM KCl by using a Chirascan CD spectrometer (Applied Photophysics, Leatherhead, UK).

### 3.6. Fibrinogen Clotting in the Presence of Aptamers

Human thrombin (50 μL, 10 U/mL) was added to a solution of fibrinogen (2 mg/mL) and aptamer (30 nM) in 1 mL of PBS in a quartz cuvette in a temperature-controlled cuvette holder of a spectrophotometer at 25 °C. Monitoring of the absorbance at 360 nm started immediately after the addition of thrombin and was stopped after the curve reached a plateau. The blank clotting curve was determined by measuring the absorbance in the absence of aptamer. The clotting curve for each sample was measured in duplicate.

### 3.7. Molecular Dynamics Simulations of TBA–OPC Complexes with Thrombin

The crystal structure of the thrombin complex with T3-modified TBA (PDB id 6z8x) was used as a starting model to build TBA OPCs. A missing thymidine residue in the TGT central loop was added to the structure. The arrangement of the added residue matched the same in the crystal structure PDB id 1hao. Then, 3D tripeptide models were attached to the terminal glycine at T3. Tripeptide models were generated using Molsoft ICM-Pro 3.9.2 [20] by creating 3D peptide models in the molecular editor followed by optimizing the conformations using molecular-mechanical and Monte Carlo approaches. To calculate interatomic interactions, the ECEPP/3 force field [21] was used at this stage. The TBA OPC models were optimized by the SYBYL X and Powell method with the following settings: partial charges and parameters for interatomic interactions were from the Amber7ff02 force field, a nonbonded cut-off distance was set to 8 Å, a distance-dependent dielectric function was applied, the number of iterations was equal to 500, the simplex method was used in the initial optimization, and the energy gradient convergence criterion of 0.05 kcal/mol/Å was applied.

The TBA OPC model stabilities were verified by MD simulations using Amber 20 software [22]. The influence of the solvent was simulated using the OPC3 water model [23]. Rectangular box and periodic boundary conditions were used in the simulation. The space between the TBA OPC model and the periodic box wall was at least 15 Å. Potassium ions were used to neutralize the negative charge of the DNA backbone and stabilize the quadruplex structure. The parameters needed for interatomic energy calculation were taken from the force fields OL15 [24,25] for the DNA and from ff14SBonlysc [26] for the peptides. At the beginning of the computation, the models of TBA OPC complexes with thrombin were optimized in two stages. First, the location of the solvent molecules was optimized by using 1000 steps (500 steps of steepest descent followed by 500 steps of conjugate gradient). At this stage, the mobility of all solute atoms was restrained by a force constant of 500 kcal×mol^−1^×Å^−2^. In the second stage, the optimization was performed without restrictions using 2500 steps (1000 steps of steepest descent and 1500 steps of conjugate gradient). Then, gradual heating to 300 K was carried out for 20 ps. To avoid spontaneous fluctuations at this point, weak harmonic restraints were applied with a force constant of 10 kcal×mol^−1^×Å^−2^ for all atoms other than the solvent ones. The SHAKE algorithm was applied to constrain hydrogen-containing bonds, which allowed the use of a 2 fs time step. Scaling of 1–4 nonbonded van der Waals and electrostatic interactions was performed by using the standard Amber values. The cut-off distance for nonbonded interactions was set to 10 Å, and the long-range electrostatics were calculated using the particle mesh Ewald method. The MD simulations in the production phase were carried out using constant temperature (T = 300 K) and constant pressure (*p* = 1 atm) over 80 ns. To control the temperature, a Langevin thermostat was used with a collision frequency of 1 ps^−1^. The energy of interaction of the amino acids in the tripeptides with thrombin was estimated using the *lie* command from the *cpptraj* module with a dielectric constant of 4. To estimate the distances between aromatic amino acids of the tripeptides, T3 nucleobase, and Tyr76 aromatics, the centers of mass of the aromatic rings were used. For the Trp residue, the shortest distance between the centers of mass of the tryptophan rings and the aromatics of the other residue was utilized. For the thrombin residues Leu65, Ile82 and Met84, the shortest distance to the carbon atoms CD1 or CD2 (in Leu65), CD1 or CG2 (in Ile82), and CE (in Met84) was considered. The plots of geometrical parameters and energy of interaction vs. time were smoothed using the moving average method (span = 5).

## 4. Conclusions

The selection of high-affinity DNA aptamers by different SELEX protocols (Systematic Evolution of Ligands by EXponential enrichment) is typically complemented by post-SELEX optimization aimed at improving aptamers’ affinity and inhibitory characteristics or resistance to biodegradation. In the current study, we demonstrate that screening aptamer OPCs is a promising strategy for post-SELEX modification. With respect to TBA, this strategy combined both screening and elements of rational design based on the structural information available for TBA–thrombin complexes. The general idea of extending the aptamer–protein recognition interface by adding a side chain to an aptamer was realized by anchoring the tripeptide subunit to the T3 nucleobase of TBA. The conjugation chemistry was based on the use of N3-modified thymidine phosphoramidite with pNP-activated carboxyl functionality. The major advantage of pNP activation over the commonly used NHS group is its notably higher hydrolytic stability, which is crucial for the preparation and purification of the building block itself and for further oligonucleotide synthesis. Although developed for the specific modification of TBA, pNP-activated thymidine phosphoramidite or its functional analogues may find wide application for the post-synthetic conjugation of oligonucleotides.

Studies of the anticoagulant characteristics of TBA–OPCs showed that the presence of the peptide side chain induced diverse effects depending on the amino acid sequence in the peptide subunit. Further molecular dynamics simulations of TBA–OPC complexes with thrombin revealed a structural basis for the observed distribution of anticoagulant activities. Amino acid 18 in the peptide side chain seems to be the most important part of the extended recognition interface. Configuration of the neighboring residues 17 and 19 may either facilitate or hinder efficient interactions between amino acid 18 and the protein residues Met84, Leu65, or Ile82. In this respect, the formation of the intramolecular π-stacking system involving the T3 nucleobase and residues 17 and 19 is unfavorable for forming new contacts with thrombin. These findings provide insights into the principles of further design of TBA–OPCs and, more generally, of other aptamer–peptide conjugates.

## Figures and Tables

**Table 1:** Characterization of TBA–OPCs.

TBA OPC	M^calc^/M^obsvd^	Y ^a^, %	*T*_m_, °C ^b^	t_1/2_, min ^c^
Unmodified TBA	4726.1/4721.9	n/a	52.5	7.1
TBA–SFN	5132.8/5138.5	47	54.2	9.6
TBA–FYW	5280.7/5284.0	66	57.9	7.6
TBA–FNW	5231.6/5241.6	63	57.5	6.5
TBA–YWN	5247.6/5250.1	72	56.8	9.8
TBA–WSY	5220.6/5222.8	70	54.9	6.7

^a^ Efficiency of conjugation (percentage of the conjugate in the total amount of full-size oligonucleotides); ^b^ in 100 mM KCl and 10 mM Na cacodylate (pH 7.4) at an oligonucleotide concentration of 5 μM; ^c^ t_1/2_ for the blank sample (without aptamer) was 1.1 min; Δ*T*_m_ = ±0.5 °C; Δt_1/2_ = ±0.1 min.

## Data Availability

Not applicable.

## Supplementary Materials

The following are available online at https://www.mdpi.com/article/10.3390/ijms23073820/s1.
