# Supplementary material for: Anticoagulant Oligonucleotide–Peptide Conjugates: Identification of Thrombin Aptamer Conjugates with Improved Characteristics
Source: Int J Mol Sci. 2022 Mar 30;23(7):3820. doi: 10.3390/ijms23073820 (PMC8998821; doi:10.3390/ijms23073820)
Supplement: Supplementary file 1 [file ijms-23-03820-s001.zip › ijms-1660175-supplementary.pdf]

Anticoagulant oligonucleotide-peptide conjugates: identification of thrombin aptamer conjugates with improved characteristics

Vladimir B. Tsvetkov [2,3], Irina V. Varizhuk [1], Nikolay N. Kurochkin [1], Sergei A. Surzhikov [1], Igor P. Smirnov [2], Andrey A. Stomakhin [1], Natalia A. Kolganova [1] and Edward N. Timofeev [1],*

[1] Engelhardt Institute of Molecular Biology, Russian Academy of Sciences, 119991 Moscow, Russia;
[2] Federal Research and Clinical Center of Physical-Chemical Medicine, 119435 Moscow, Russia;
[3] Institute of Biodesign and Complex System Modeling, Sechenov First Moscow State Medical University, 119146 Moscow, Russia

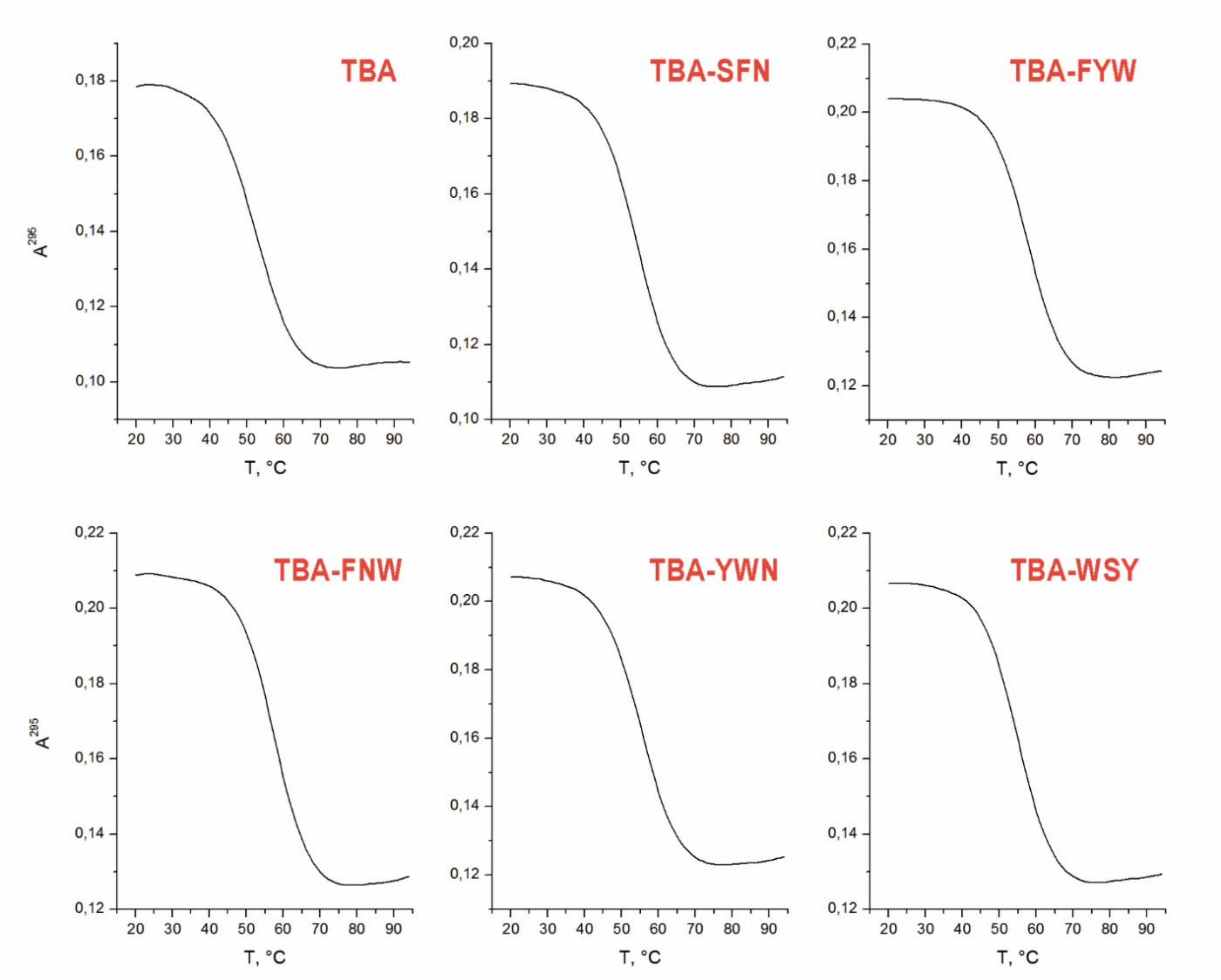

Figure S1. Melting profiles of TBA OPCs (295 nm) in 10 mM sodium cacodylate (pH 7.2) and 100 mM KCl at an oligonucleotide concentration of 5 μM

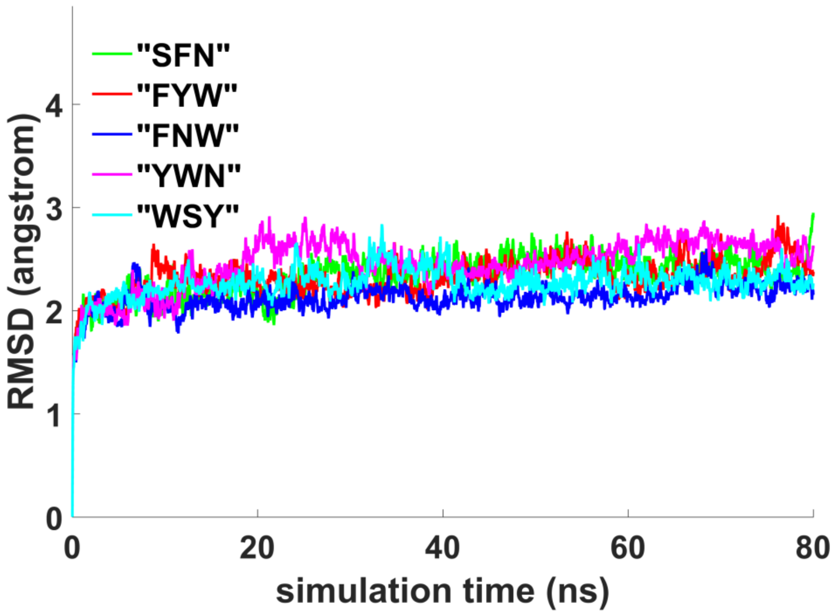

Figure S2. RMSD data for TBA-OPC complexes with thrombin.

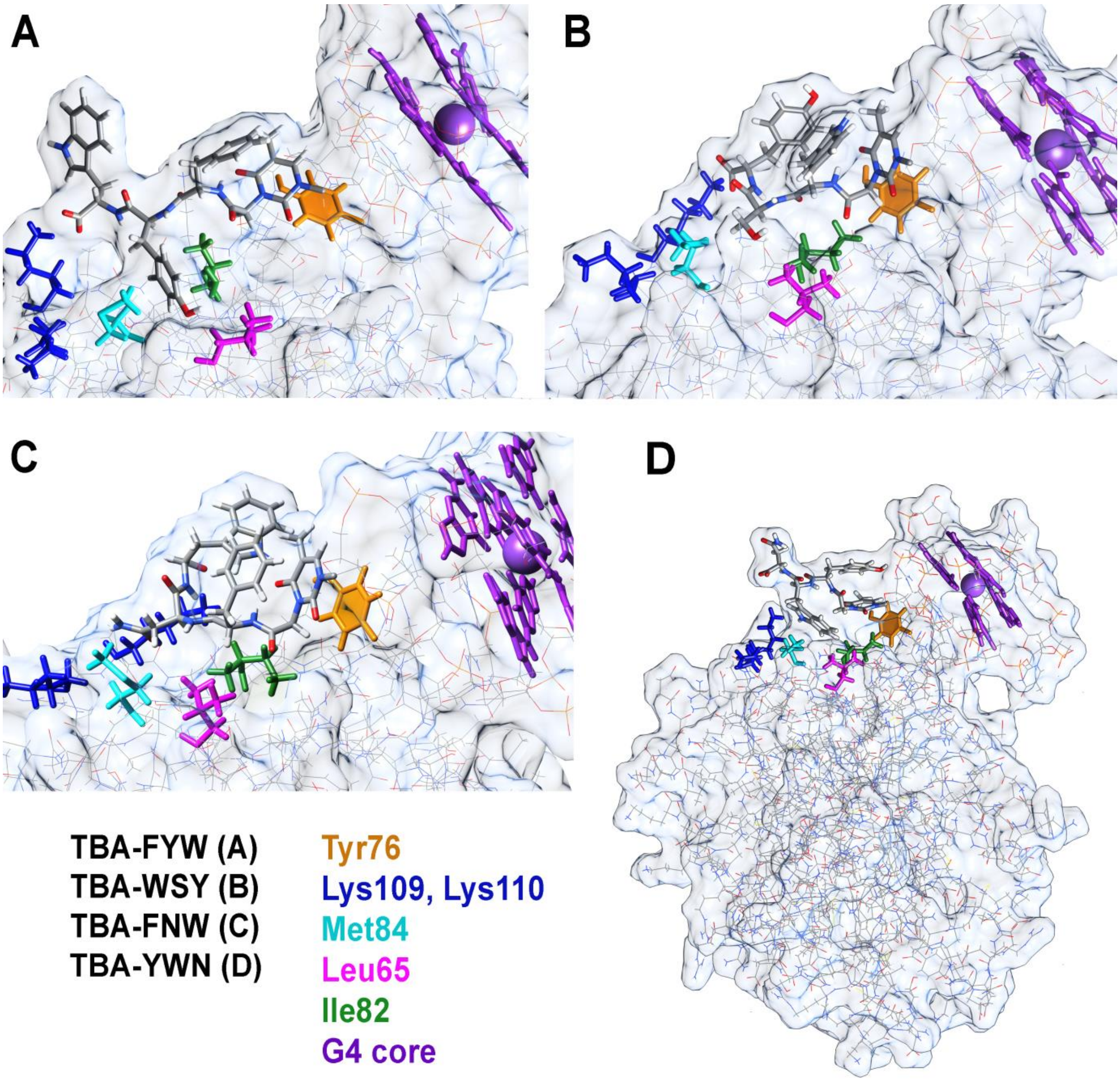

Figure S3. Snapshots of thrombin complexes with TBA-FYW, TBA-WSY, TBA-FNW, and TBA-YWN (full size) at 80 ns.

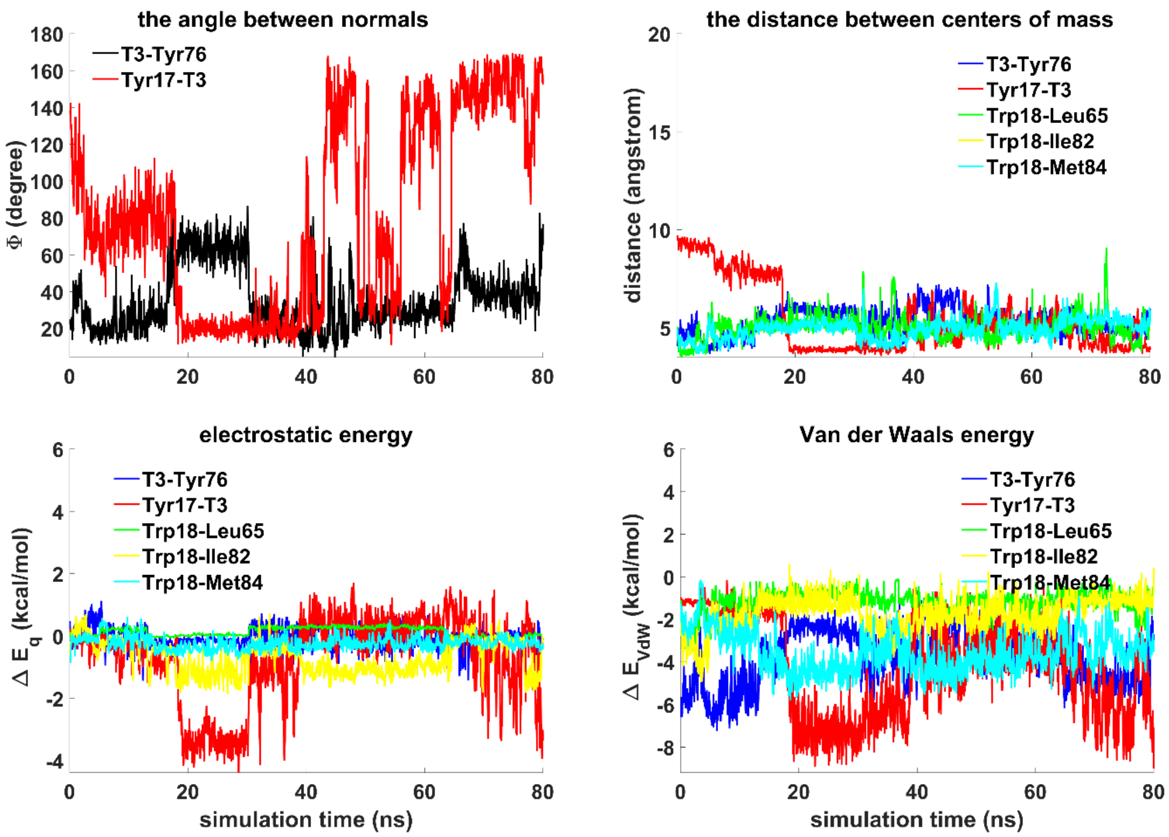

Figure S4. Geometrical parameters and energy of interaction between selected residues for TBA-YWN complex with thrombin.

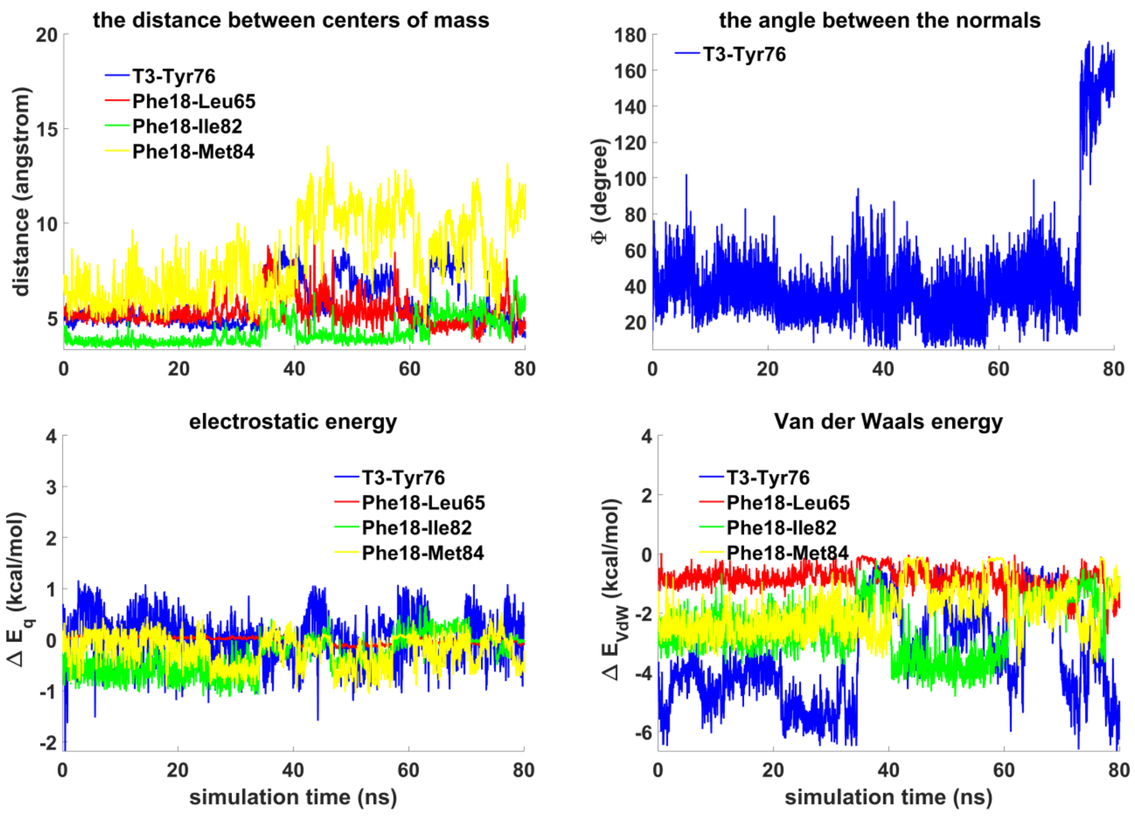

Figure S5. Geometrical parameters and energy of interaction between selected residues for TBA-SFN complex with thrombin.

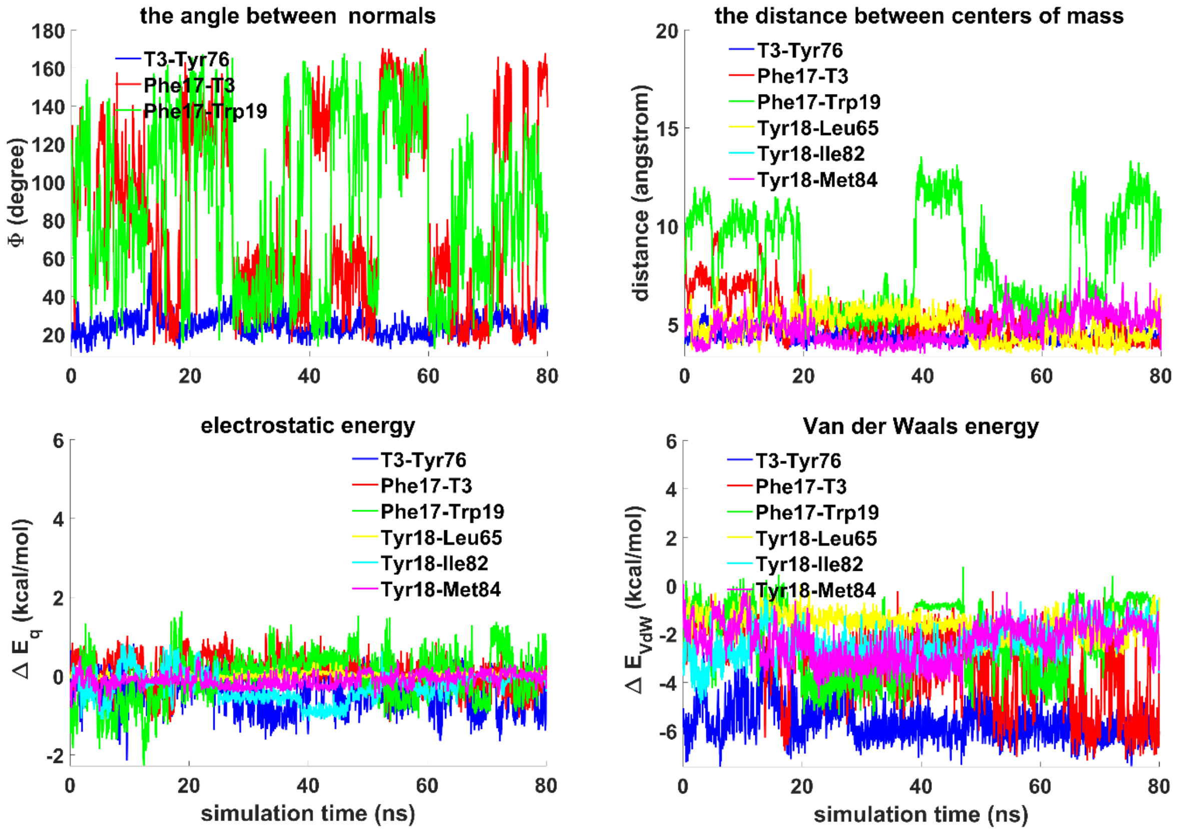

Figure S6. Geometrical parameters and energy of interaction between selected residues for TBA-FYW complex with thrombin.

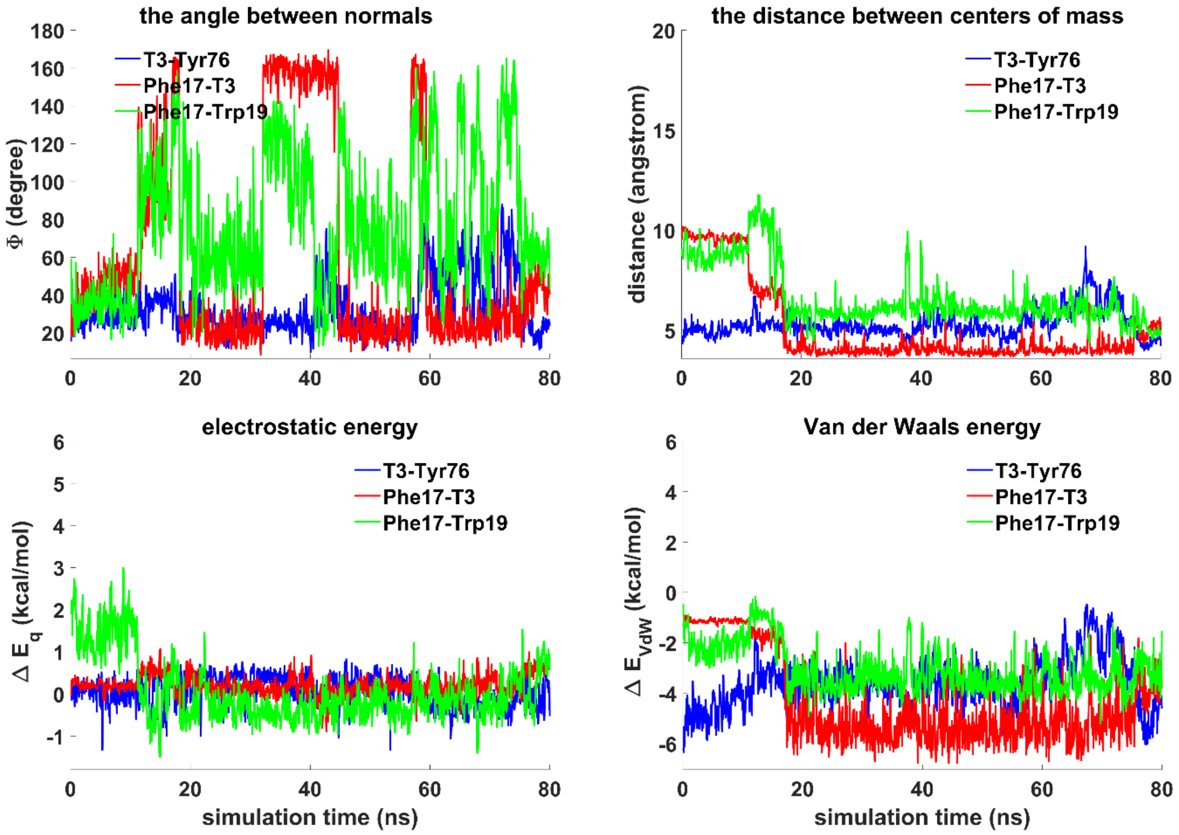

Figure S7. Geometrical parameters and energy of interaction between selected residues for TBA-FNW complex with thrombin.

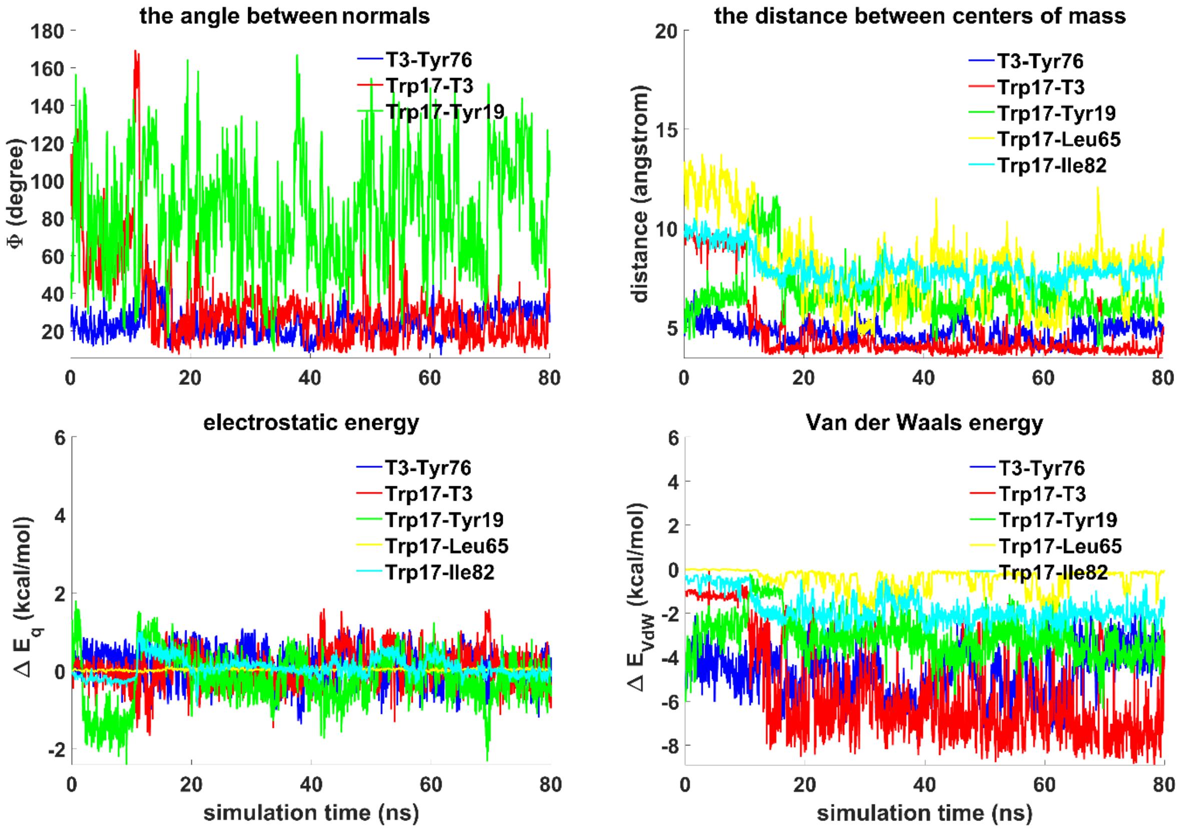

Figure S8. Geometrical parameters and energy of interaction between selected residues for TBA-WSY complex with thrombin.

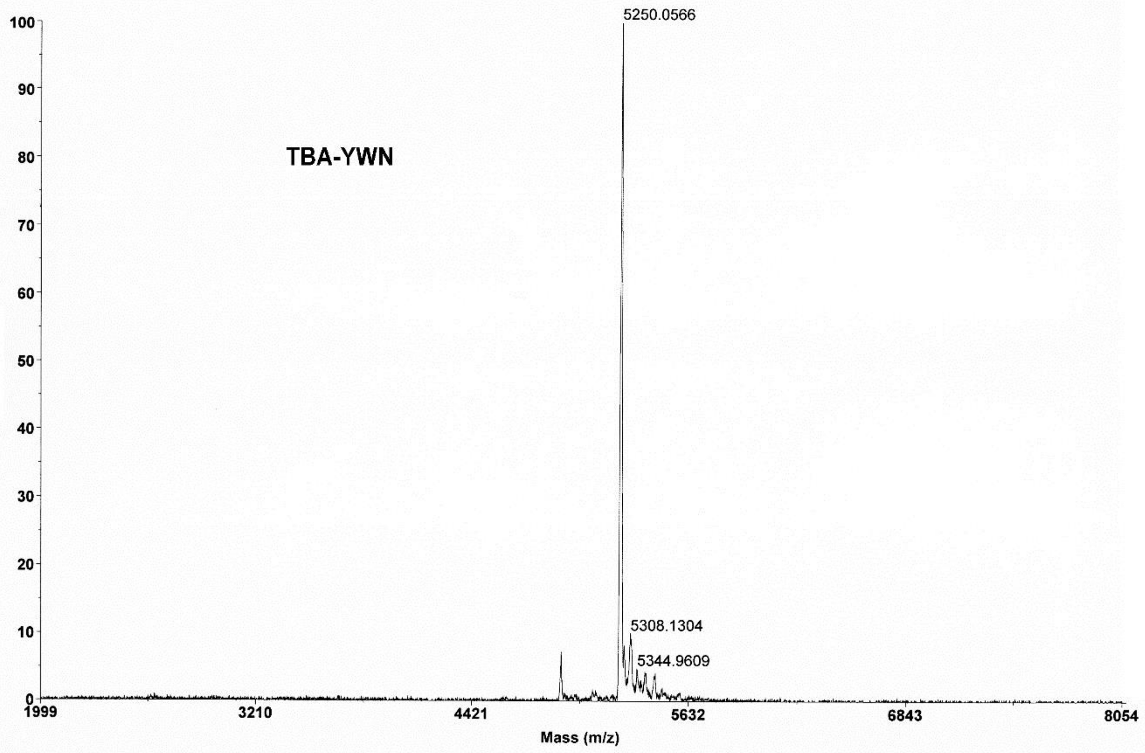
5250.0566
TBA-YWN
5308.1304
5344.9609
100
90
80
70
60
50
40
30
20
10
0
1999
3210
4421
5632
6843
8054
Mass (m/z)

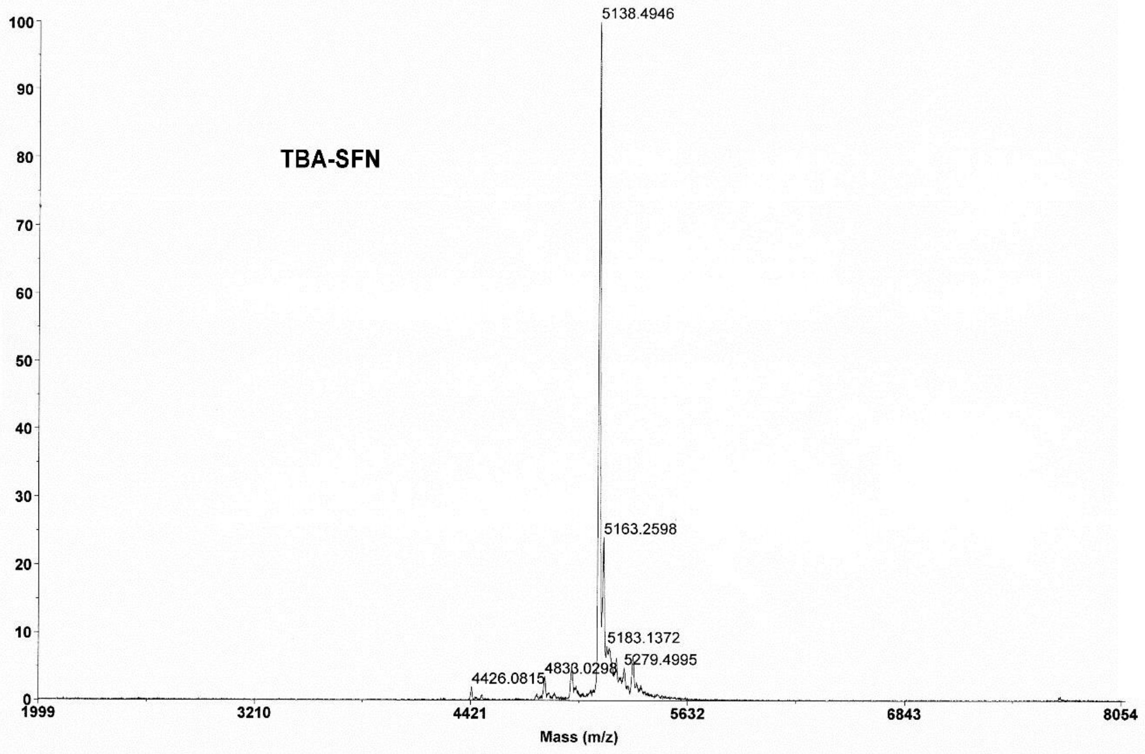
5138.4946
TBA-SFN
5163.2598
5183.1372
5279.4995
4426.0815
4833.0298
100
90
80
70
60
50
40
30
20
10
0
1999
3210
4421
5632
6843
8054
Mass (m/z)

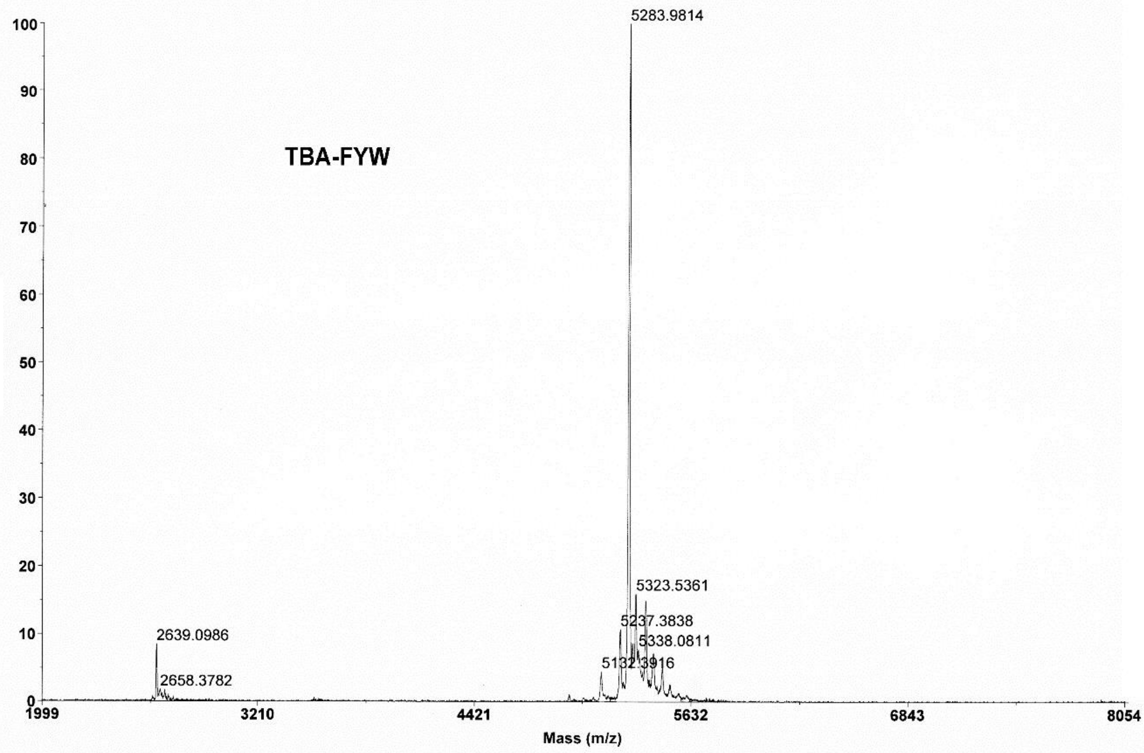
TBA-FYW
5283.9814
5323.5361
5237.3838
5338.0811
5132.3916
2639.0986
2658.3782
100
90
80
70
60
50
40
30
20
10
0
1999
3210
4421
5632
6843
8054
Mass (m/z)

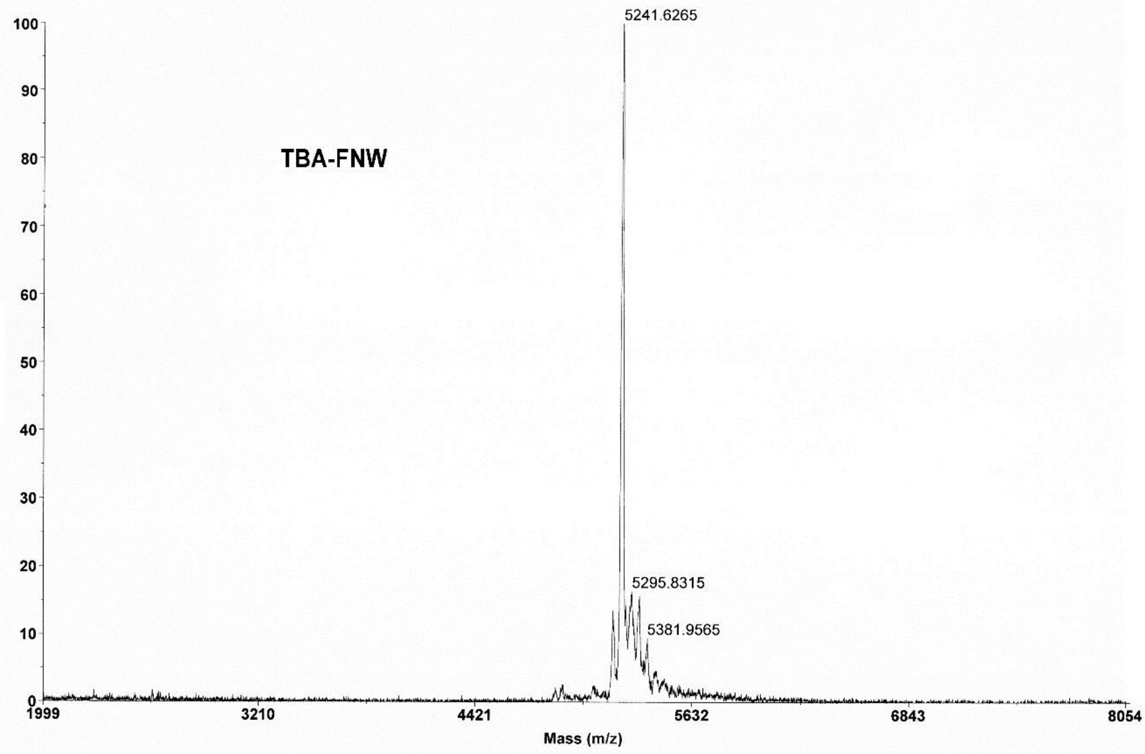
TBA-FNW
5241.6265
5295.8315
5381.9565
100
90
80
70
60
50
40
30
20
10
0
1999
3210
4421
5632
6843
8054
Mass (m/z)

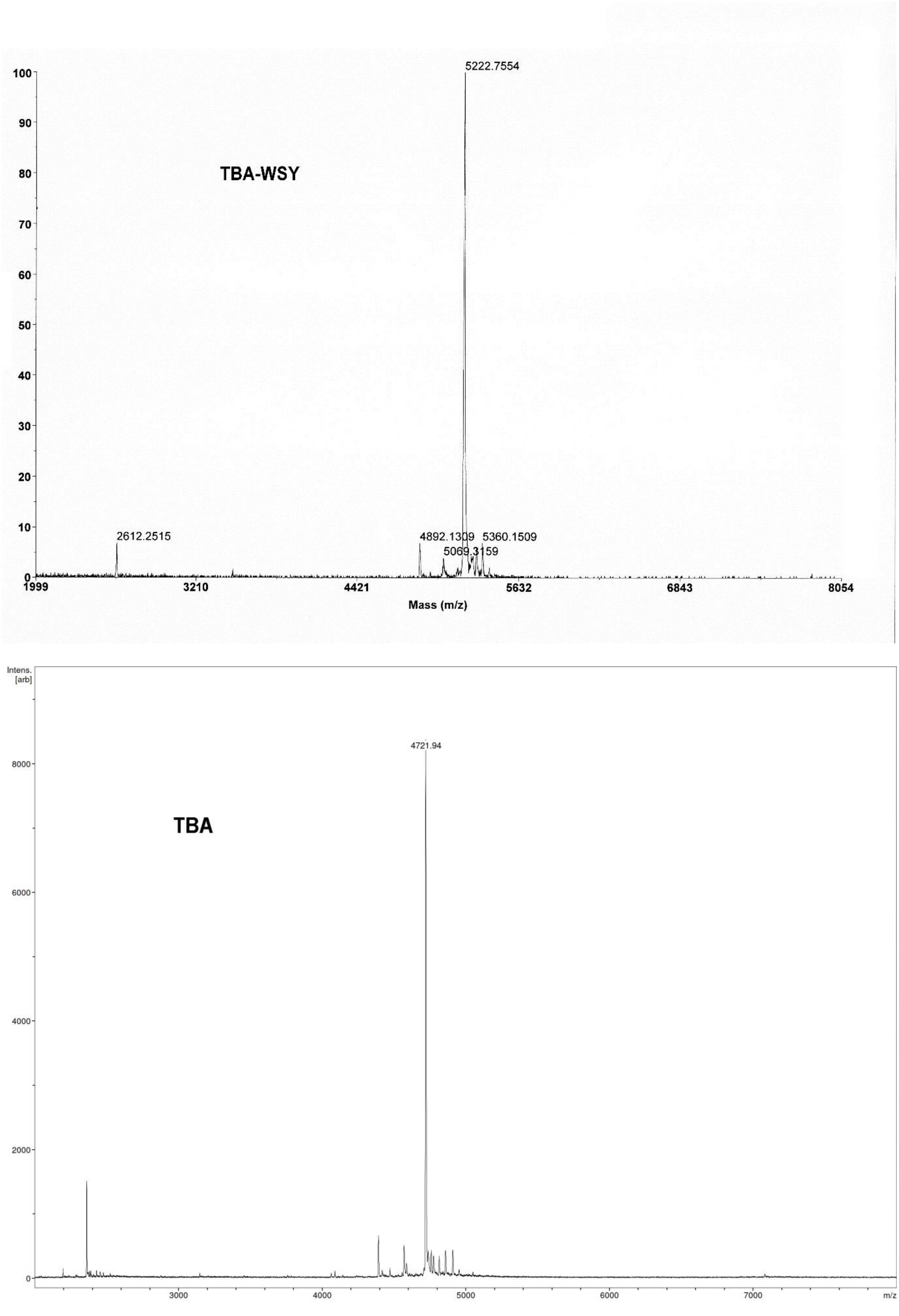

Figure S9. MALDI mass spectra of TBA OPCs.
